# Enhancing the potential of rapeseed cake as protein-source food by γ-irradiation

**DOI:** 10.1042/BSR20231807

**Published:** 2024-03-13

**Authors:** Chuan Xiong, Xin Zou, Chia-Wei Phan, Wenli Huang, Yu Zhu

**Affiliations:** 1 Biotechnology and Nuclear Technology Research Institute, Sichuan Academy of Agricultural Sciences, Chengdu 610061, China; 2 College of Food and Biological Engineering, Chengdu University, Chengdu 610106, China; 3 Department of Pharmaceutical Life Sciences, Faculty of Pharmacy, Universiti Malaya, Kuala Lumpur 50603, Malaysia

**Keywords:** γ-irradiation, Acute-toxicity test, Antinutritional factors, By-product, Rapeseed protein

## Abstract

Rapeseed cake serves as a by-product in the oil extraction industry, characterized by its elevated protein content. However, the presence of antinutritional factors limits the utilization of rapeseed cake as a viable protein source. In this study, different doses of γ-irradiation were used to irradiate rapeseed cake and rapeseed protein isolate was extracted through a modified alkaline solution and acid precipitation method from rapeseed cake. The chemical composition and *in vivo* acute toxicity of rapeseed protein isolate were determined. The protein recovery rate of rapeseed protein isolate was 39.08 ± 3.01% after irradiation, while the content of antinutritional factors was significantly reduced. Moreover, γ-irradiation did not have any experimentally related effects on clinical observations or clinicopathology in mice. Overall, the reduced antinutrients and increased functional properties suggest that the irradiation of rapeseed cake (<9 kGy) could be utilized as a pre-treatment in the development of rapeseed cake-based value-added protein products.

## Introduction

Rapeseed (*Brassica juncea* L., *Brassica rapa* L., and *Brassica napus* L.), an herbaceous and annual plant classified within the Brassicaceae family, is predominantly cultivated for its oil-rich composition. With its >40% oil content, rapeseed is the third most cultivated oil crop globally [1]. It is worth noting that in addition to the high oil content, the protein content of rapeseed cake (RSC) is also high (35–40% of dry matters) [2]. However, the process of extracting the rapeseed oil results in a currently underutilized by-product of a protein-rich meal or press cake. In the year 2019–2020, approximately 69 million tons of rapeseeds were harvested worldwide, and over 39 million tons of residues were produced after mechanical oil pressing [3]. Previous studies have suggested the necessity of utilizing the solid wastes of edible oil production due to abundance of nutrients and bioactive compounds in these materials [4].

As a promising source of dietary protein due to its well-balanced amino acid composition and higher proportion of S-amino acids as compared to many other plants, rapeseed is a valuable source of protein for human consumption and even as animal food [5]. Despite this, antinutritional factors (ANFs) limit the utilization of rapeseed protein. Glucosinolates (GSL), phytates (also known as phytic acid), and phenolics are common ANFs. High-ANFs rapeseed meals or cakes used as animal food are often accompanied by adverse effects, such as kidney and liver dysfunctions, antithyroid effect, iodine deficiency, and pungency of isothiocyanates [6]. Specifically, a diet with high glucosinolate content will affect the thyroid function of animals, leading to maternal malnutrition, and ultimately lead to maternal reproductive disorders [7]. Phytic acid has a strong chelating effect on minerals and proteins, which leads to weak digestion and absorption of proteins and minerals in monogastric animals [8]. In addition, studies have confirmed that high content of phenolic compounds will also reduce the bioavailability of protein [9]. Due to the negative impact of ANFs, ANFs in rapeseed should be reduced to an appropriate amount before being used as protein-rich food for human consumption or directly fed to animals.

The removal or reduction of ANFs from rapeseed meals or cakes has been attempted in several ways. Some rape varieties with low glucosinolate content have been selected and popularized. Canola (*Brassica napus*) is usually referred to as ‘double-zero rapeseed’ as the edible oil obtained by pressing or extracting the seeds of canola contains <2% of erucic acid. In its meal residue, the content of GSL is <30 μmol/g [10]. However, as compared with the large-scale planting in Europe and North America, the planting area of canola in China is not large due to the natural climate, soil conditions and dietary habit. At present, rape with high glucosinolate content is still planted in large areas in China, thus, improved physicochemical methods to reduce/remove glucosinolate content are still needed. An important step in the conversion of rapeseed residues to useful products of higher value has been the pretreatment step(s), which have been designated as a key stage in upgrading rapeseed residues [9]. In addition, the pretreatment stage must be simple and practical to meet the requirements of reducing or removing multiple ANFs. The various methods proposed earlier to detoxify rapeseed include chemical treatments with alkali, NH^4+^, Fe^2+^, Ca^2+^ or salts, fermentation, or heat treatments, have not been commercially exploited or scaled up to practice, largely due to incomplete removal, excessive water consumption, high cost or loss of useful soluble proteins [11,12]. This makes it very difficult and urgent to find a satisfactory method to remove or reduce ANFs in rapeseed residues and improve their nutritional value.

The use of irradiation treatment to preserve raw and processed foods and agricultural commodities has been well established around the world as a method to prolong their shelf life or improve their hygienic qualities. Food and agricultural products can be efficiently decontaminated and disinfested through radiation processing [13]. It has been proven that some anti-nutrients in cereals and legumes are reduced or eliminated by radiation alone or in combination with other processing methods [14]. Moreover, according to the FAO/IAEA/WHO joint expert committee, food commodities irradiated up to 10 kGy are not toxicologically dangerous [15]. Therefore, the research on the removal of ANFs by irradiation technology is promising.

The mechanism of the removal of ANFs by irradiation still has many gaps, and the safety of irradiated RSC has not been systematically evaluated. Therefore, in this study, our aim is to reduce and remove ANFs from RSC and improve the nutritional value of RSC by γ-irradiation. To achieve this purpose, the effects of different doses of γ-irradiation on the contents of GSL, phytic acid, and phenolic substances in rapeseed protein were studied. In addition, we also conducted a 14-day acute toxicity test on the extracted rapeseed protein in mice to determine the toxicity of these protein products and provide information for the research and development of rapeseed protein products as animal feed or human consumption.

## Materials and methods

### Materials and chemicals

Rapeseed (*B. juncea*) cake (RSC) used in the present study was obtained from the seed station of the Sichuan Academy of Agricultural Sciences (Chengdu, Sichuan, CHN). To achieve a constant weight, RSC was dried in an oven at 37°C for a period of 24 h. Then, the obtained dry RSC was ground to pass a 60-mesh size sieve, and the obtained powder was placed in a 4°C refrigerator for later use. Citric acid (CAS 77-92-9), sodium hydroxide (CAS 1310-73-2), and sodium chloride (CAS 7647-14-5) were obtained from Merck (Darmstadt, GER). All solvents and reagents were analytical reagent grade.

The mice employed in the present study were sourced from Dashuo Experimental Animal Co., Ltd. (Chengdu, Sichuan, CHN) and were obtained under the production license No. SCXK (Sichuan) 2015-030. The conventional mouse feed, adhering to the national standard (GB14925-2010), was procured from Huafukang Biotechnology Co., Ltd. (Chengdu, Sichuan, CHN). The experimental procedures were performed in accordance with the National Medical Products Administration (NMPA, CHN) for animal experimentation and approved by the institutional animal ethics committee (Ref. no. SYXK [Chuan] 2015-197). Animal feeding and subsequent acute toxicity experiments on mice were completed at the Analysis and Testing Center of West China School of Public Health, Sichuan University (Chengdu, CHN).

### Irradiation treatment

An irradiation experiment with different doses of γ-irradiation was conducted on RSC powder packed in polyethylene bags. The weight of RSC powder in each polyethylene bag was set as 50 g, and the irradiation dose was set as 3, 5, 7, and 9 kGy. A cobalt-60 irradiator (Nordion, CAN) at Biotechnology and Nuclear Technology Research Institute (Chengdu, Sichuan, CHN) was selected as irradiation equipment and the dose rate was set as 5 kGy/h. In the present study, the RSC after various doses of irradiation was named as rRSC-1, rRSC-2, rRSC-3, and rRSC-4, respectively.

### Physicochemical characteristics of RSC and rRSCs

#### Morphology observation

The morphology of RSC and rRSCs was observed using a scanning electron microscope (SEM). RSC powder was dispersed into anhydrous ethanol, the obtained suspension was then dispersed drop by drop on the conductive film. With a sputter coater (Q150R Plus, Quorum, U.K.) under vacuum, gold was sputtered onto the surface followed by microscopic examination with a SEM (JSM-7500F, JEOL, JPN). The appropriate resolution was selected to take images on the RSC surface. The process was repeated for rRSCs.

#### Fourier transforms infrared (FTIR) spectroscopy

RSC powder was mixed with KBr, dried at 110°C for 48 h, and then compressed with tablet press under 6 MPa pressure. The mixed powder was placed in the instrument, vacuumized, the infrared background peak was deducted, and then the infrared interference spectrum of the material was measured (Nicolet 6700, Thermo Electron Corporation, U.S.A.). The interference data were calculated by Fourier transform with computer software (Omnic 8.0, Nicolet, U.S.A.), and the frequency spectrum with wavelength and transmissivity as functions was obtained. The process was repeated for rRSCs.

### Preparation of RPI and irradiated-RPI (rRPI)

Alkali dissolution and acid precipitation method was used to prepare RPI and rRPI. Briefly, RSC powder and distilled water were mixed in a mass volume ratio of 1:5 and stirred with a magnetic stirrer (MS5, JONALAB, CHN) for 1 h. The 1 M sodium hydroxide solution was used to adjust the pH of RSC suspension to 10.5 and placed in a 4°C refrigerator for 3 h. The suspension was separated by centrifugation at 10000 × ***g*** for 20 min (Allegra V-15R, Beckman Coulter, Indianapolis, U.S.A.). Then the supernatant was collected, and pH was adjusted to 5.0 with citric acid with a molar concentration of 2 M. Then we centrifuged the dispersion at 5000 × ***g*** for 30 min in 4°C and collected the precipitate. The precipitate was placed in the oven, dried by hot air at 37°C for 6 h and RPI was the collected dry precipitate. The rRPI was obtained from rRSC powder by applying the same method as RPI.

### Chemical composition of RSC, rRSC, RPI, and rRPI

The determination of moisture, ash, and crude fat contents was conducted in accordance with the method prescribed by the Official Society of Agricultural Chemists. The determination of crude protein content for both RSC and RPI was completed by Dumas method. The nitrogen content of protein was measured by using a Flash EA 1112 system (Thermo, MA, U.S.A.). Crude protein content in each sample was calculated using a protein factor of 6.25 to convert the nitrogen content to protein content. Crude protein recovery was calculated as followed:

$$Crude\;protein\;recovery\;yield\;\left(\%\right)=\frac{m_{1}\times a}{m_{2}\times b}\times 100\%$$

In this equation: *m*_1_ represents RPI mass (g), *a* represents the protein content rate of RPI (%), *m*_2_ represents RSC mass (g) used in one test, and *b* represents the protein content rate of RSC (%).

Similarly, the aforementioned method was applied for the evaluation of antinutritional compounds. Total phenolics (TP) were quantified by employing the Folin-Ciocalteu reagent, with sinapic acid serving as the standard compound, as elucidated in the similar work of Szydowska-Czerniak et al. The quantification of the glucosinolate content in the samples was accomplished through the utilization of a palladium chloride spectrophotometric method. Phytic acid levels were assessed using a colorimetric molybdenum-blue assay, following the meticulously described protocol outlined in the research conducted by McKie et al [16].

### Sodium dodecyl sulphate-polyacrylamide gel electrophoresis (SDS-PAGE)

The SDS-PAGE experiment was completed according to the method of Grudniewska et al., with some minor modifications [17]. Briefly, 1 mg of RPI powder was accurately weighed and dissolved in 1 ml of distilled water. A total of 50 μl of the solution obtained by dissolution was measured and mixed evenly with 200 μl of sample buffer solution (P0015, Beyotime, Shanghai, CHN). Then, the mixture was placed in a boiling water bath for 10 min. An aliquot (20 μl) of protein standard markers and the prepared samples were loaded on to the gel (5% stacking gel and 15% separating gel). The voltage of gel electrophoresis was set to 80 V and Coomassie blue fast staining solution was used to dye the gel in the experiment, and then wash it with distilled water.

### Animal experiments

The acute oral toxicity test was conducted according to the procedures outlined by the Organization for Economic Co-operation and Development (OECD) 423 [18,19]. Seventy adult C57 mice (weighing 20 ± 2 g) were randomly divided into seven groups, each consisting of 10 mice (five males and five females): the control group (CK), RPI (20 g/kg) group, rRPI (20 g/kg) group, and soya bean protein isolate group (SPI; 20 g/kg). Among them, the rRPI group (20 g/kg) was further divided into four groups based on different irradiation doses, named rRPI-1, rRPI-2, rRPI-3, and rRPI-4, respectively. Prior to administration, a 12-h fasting period was observed, with ad libitum access to water. The test samples were prepared by dissolving the respective substances in deionized water, following which they were administered to the mice via gavage. The CK received an equivalent volume of deionized water as a single administration. Over a duration of 14 days, the mice were closely monitored for any signs of adverse effects, including alterations in behavior and skin coloration. Daily recordings of body weights were conducted to assess potential fluctuations. At the end of the 14-day acute toxicity test, the mice were anesthetized using pentobarbital sodium. Blood samples were collected from the different groups for subsequent analysis. Serum levels of aspartate aminotransferase (AST) and alanine aminotransferase (ALT) were quantified using commercially available kits supplied by Beyotime (Shanghai, CHN). Hematological parameters and the level of thyroid-releasing hormone (TRH) were determined using a kit obtained from Hermes Criterion Biotechnology (Vancouver, Canada). Carbon dioxide euthanasia was employed to ensure a humane and ethical approach, and vital organs, including the heart, liver, spleen, kidneys, thyroid gland, parathyroid glands, ovaries, uterus, testes, and prostate gland, were meticulously collected for subsequent histopathological examination.

### Statistical analyses

All independent experiments were performed three times in the present study. Data were presented as mean ± standard deviation (SD). Checking for data normality was carried out using the Shapiro–Wilk test, with *P-*value > 0.05 indicating a normally distributed continuous variable. The significance of differences between the groups was determined by a one-way analysis of variance (ANOVA) using SPSS 19.0 software (SPSS Inc., Chicago, IL, U.S.A.). As a result of Duncan’s multiple range test (DMRT), significance was determined at *P*<0.05 in all experiments.

## Results

### Morphology observation of RSC and rRSCs

**Figure 1:**
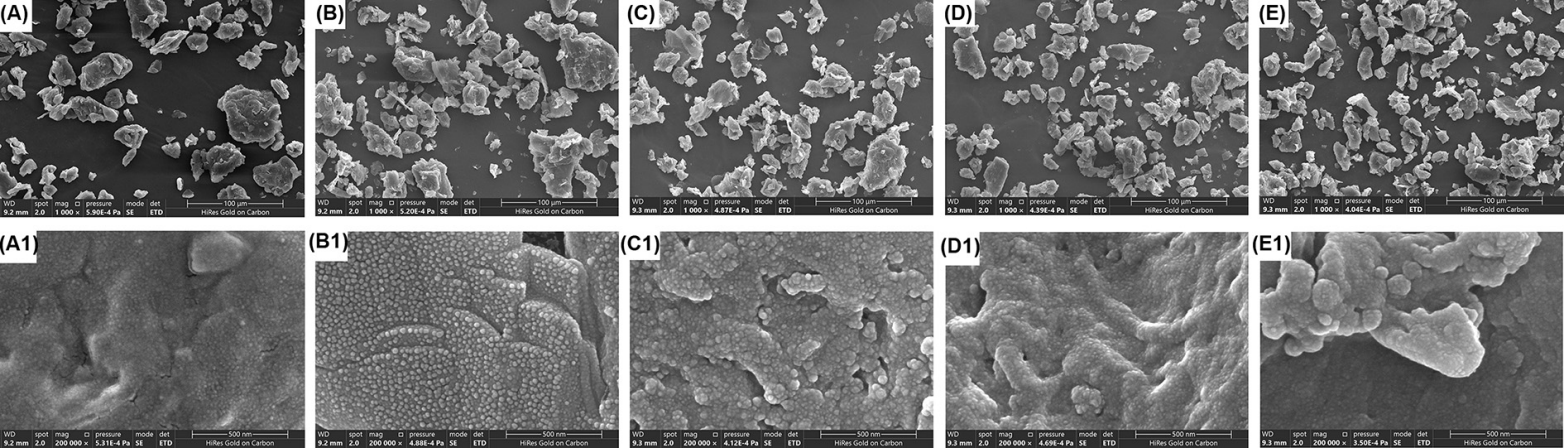
Morphology of RSC and rRSCs (**A**) SEM images for RSCs, (**B–E**) SEM images for rRSCs, and a dose of 3, 5, 7, and 9 kGy was used in the irradiation process, respectively. SEM observation magnification was set to 1000×. (A1–E1) In accordance with the SEM images of (A–E), the magnification has been set to 2,000,000×.

RSC powder that has been dried in a 37°C oven for 24 h and passed through a 60-mesh sieve exhibited a granular structure under SEM (Figure 1A). At higher magnification, the surface of the granular structure was rough and flaky (Figure 1A1). The irradiation treatment at a dose of 3 kGy resulted in a smaller granular structure and roughened surface of RSC (Figure 1B), accompanied by a large number of warty protrusions (Figure 1B1). As the irradiation dose increased, the granular structure of RSC continued to decrease and became more uniform, but the warty protrusions on the surface decreased and the surface became relatively smooth.

### FTIR analysis

**Figure 2:**
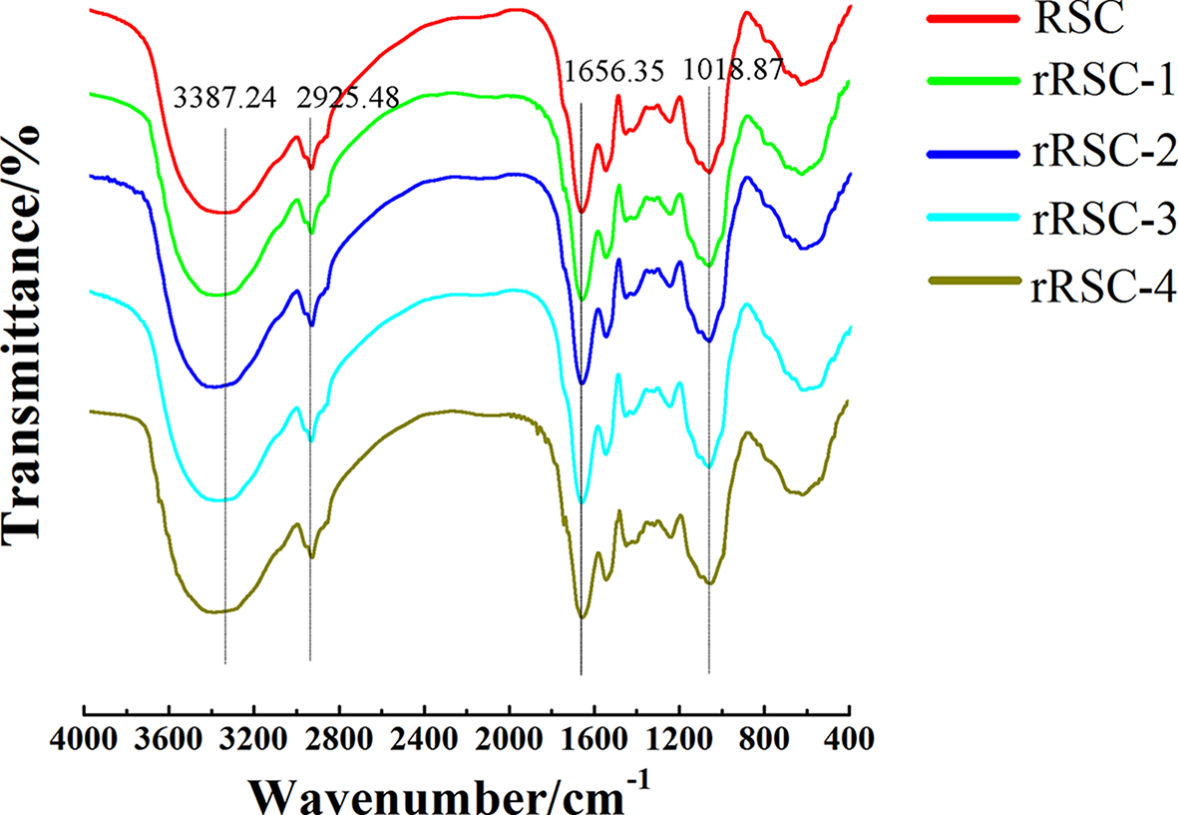
The FTIR-spectra of RSC and rRSCs

Figure 2 illustrates the infrared spectrums of RSC and rRSCs. In the IR spectra of the samples, no new peaks were observed in both RSC and rRSCs, which confirmed that no new compounds were formed during the irradiation process. In the infrared spectrum, the main absorption peaks observed correspond to wavenumbers of 1018, 1656, 2925, and 3387 cm^−1^, respectively. As compared with the natural sample (RSC), the change in peak at 1014 cm^−1^ in the irradiated sample was attributed to changes in C-O-H, which confirmed that the ordered structure of the sample had been reduced. This was consistent with the SEM results. As a result of stretching O–H, the intense broadband at 3387 cm^−1^ were observed. Compared with RSC in the present study, the rRSCs’ hydroxyl absorption peak had a substantial shift in wave number. This change was directly related to the hydrogen bonding and water content in the sample, which can further confirm the changes in protein recovery rate, phenolics, and phytic acid in the irradiated sample.

### Chemical composition of RSC, rRSC, RPI, and rRPI

Protein, fat, and ash content in RSC and rRSCs did not significantly change under the irradiation dose used in this experiment, as shown in Table 1. However, when the irradiation dose reached 9 kGy, the crude fiber content in RSC (rRSC-4) was significantly reduced. The alkali-dissolution-acid precipitation method was used for the isolation of proteins from RSC and rRSC, named RPI and rRPI. There had been a significant improvement in the protein content of RPI as compared with RSC, and irradiation treatment had further strengthened this trend. rRPI-4 exhibited the highest protein content, reaching 88.38 ± 3.92%. Fat, ash, and crude fiber content in RPI and rRPI changed in the opposite direction from RSC and rRSC, and all decreased significantly. A significant increase in the recovery yield of protein from RSC can be achieved by irradiation, which can increase the recovery yield from alkali-dissolving and acid-precipitation methods to 39.15 ± 2.92%.

**Table 1:** Chemical composition of RSC, rRSC, RPI, and rRPI

Chemical composition	RSC	rRSC-1	rRSC-2	rRSC-3	rRSC-4	RPI	rRPI-1	rRPI-2	rRPI-3	rRPI-4
**Protein (%)**	38.14 ± 0.93^§^	38.04 ± 0.55§	38.12 ± 0.76§	38.33 ± 0.44§	38.02 ± 0.92§	76.65 ± 2.76‡	84.41 ± 3.35†	84.26 ± 3.11†	87.26 ± 4.41*	88.38 ± 3.92*
**Fat (%)**	33.53 ± 1.15^*^	33.47 ± 0.96*	33.63 ± 0.89*	32.52 ± 0.82*	32.53 ± 0.71*	1.68 ± 0.21†	1.34 ± 0.19‡	1.32 ± 0.23‡	1.17 ± 0.16§	1.16 ± 0.20§
**Ash (%)**	7.83 ± 0.31^*^	7.81 ± 0.53^*^	7.76 ± 0.34^*^	7.71 ± 0.32^*^	7.78 ± 0.27^*^	5.58 ± 0.89^†^	2.64 ± 0.47^‡^	2.71 ± 0.52^‡^	2.58 ± 0.34^‡^	2.63 ± 0.31‡
**Crude fiber (%)**	15.25 ± 0.63^*^	15.63 ± 0.47^*^	15.98 ± 0.62^*^	15.17 ± 0.32^*^	14.12 ± 0.55^b^	0.82 ± 0.13^‡^	0.79 ± 0.10‡	0.77 ± 0.17^‡^	0.78 ± 0.09‡	0.79 ± 0.12^‡^
**Protein Recovery Yield (%)**	ND	ND	ND	ND	ND	32.23 ± 1.48^‡^	36.63 ± 1.92^†^	36.04 ± 2.02^†^	39.15 ± 2.92*	39.08 ± 3.01^*^
**Glucosinolates (μmol/g)**	68.82 ± 2.54*	33.38 ± 2.91†	32.25 ± 1.77†	15.54 ± 2.05^‡^	14.41 ± 1.12‡	32.56 ± 1.94^‡^	4.27 ± 0.78^§^	4.05 ± 0.69^§^	1.78 ± 0.23^║^	1.92 ± 0.15^║^
**Phytic acid (%)**	4.65 ± 0.56^*^	3.57 ± 0.48^†^	3.41 ± 0.33^†^	3.32 ± 0.17†	3.01 ± 0.16^†^	0.24 ± 0.08^‡^	0.22 ± 0.02^‡^	0.21 ± 0.03^‡^	0.21 ± 0.03^‡^	0.20 ± 0.02^‡^
**Phenolics (%)**	2.64 ± 0.36^*^	1.75 ± 0.29^†^	1.74 ± 0.23†	1.69 ± 0.21^†^	1.70 ± 0.20†	0.31 ± 0.09^‡^	0.29 ± 0.04‡	0.29 ± 0.05^‡^	0.30 ± 0.03‡	0.28 ± 0.02^‡^

ND, Not detected. RSC, RSC powder that has been dried in a 37°C oven for 24 h and passed through a 60-mesh sieve. rRSC-1, rRSC-2, rRSC-3, and rRSC-4: RSC powder obtained after different doses of irradiation treatment, the irradiation dose was sequentially set as 3, 5, 7, and 9 kGy. RPI, Protein isolate extracted from RSC, using alkaline solution and acid precipitation method. rRPI-1, rRPI-2, rRPI-3, and rRPI-4: Protein isolate extracted from rRSC-1, rRSC-2, rRSC-3, and rRSC-4, respectively. The data were presented with mean ± SD, and each independent experiment was repeated three times. Different symbols of data in the same row represent significant differences at the 5% level (*P*<0.05).

Both alkaline solution and acid precipitation extraction and irradiation treatment can effectively reduce the content of anti-nutritional factors (ANFs) in RSC. RSC glucosinolate content was reduced by 50% by low-dose irradiation treatments (3 and 5 kGy), which was comparable with alkali soluble acid precipitation. This value was nearly 75% in the high-dose irradiation treatment group (7 and 9 kGy). Moreover, GSL were also basically removed by alkali dissolution and acid precipitation after irradiation. A similar trend was observed in the removal of phytic acid and phenolics.

### SDS-PAGE

**Figure 3:**
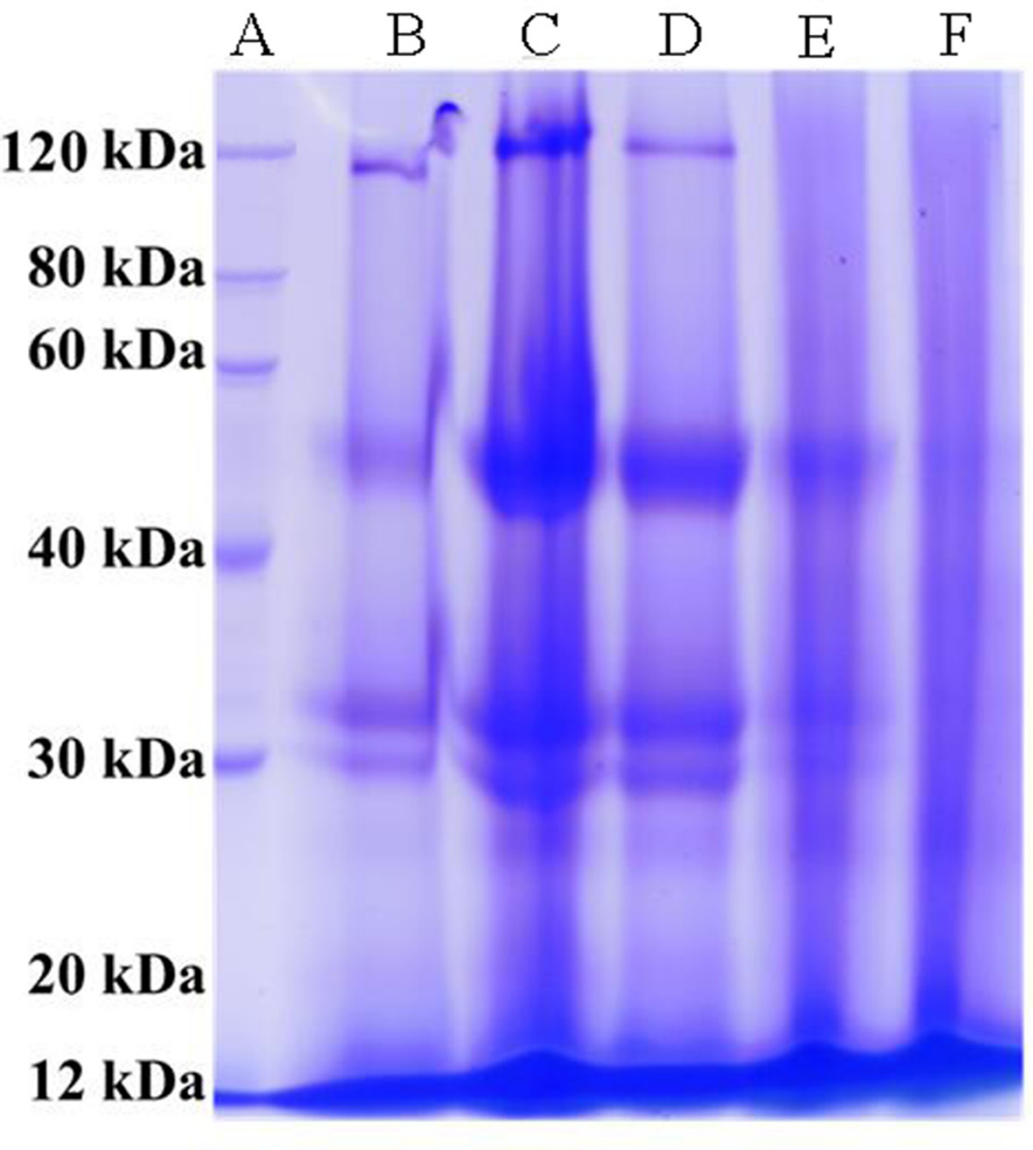
SDS-PAGE of RPI and rRPIs Column A: Standard markers; Column B: RPI, protein isolate extracted from RSC, using alkaline solution and acid precipitation method; Column C-F: rRPI-1 to rRPI-4, protein isolate extracted from rRSC-1, rRSC-2, rRSC-3, and rRSC-4, respectively. rRSC means RSC powder obtained after different doses of irradiation treatment.

An SDS-PAGE analysis was carried out to determine the molecular weights of proteins in the RPI and rRPI fractions. There were proteins with a relative molecular weight range of 12–120 kDa in RPI, and the highly concentrated bands were located approximately 30, 35, 50, and 120 kDa. A similar set of bands was found on rRPI-1 (Figure 3, Column C) and rRPI-2 (Figure 3, Column D). However, rRPI-3 (Figure 3, Column E) did not have the band approximately 120 kDa as observed in rRPI-1 and rRPI-2. Moreover, for rRPI-3 (Figure 3, Column E), the band approximately 50 kDa showed a weakening trend and unseparated bands (indistinguishable units) at 12‒30 kDa. A similar result was observed in rRPI-4 (Figure 3, Column F).

### Acute toxicity of RPI and rRPI *in vivo*

A normal growth trend was seen within 14 days of administration for the seven groups of mice that were tested for acute toxicity (Supplementary Figure S1). In addition, no significant abnormalities were observed in the skin color and behavior of the test mice during the experiment. A routine blood test of mice fed with RPI showed that they had a significant increase in white blood cells and a significant decrease in lymphocytes as compared with the control mice. rRPI-1-administered mice also demonstrated this change in their blood. However, other treated mice (rRPI-1, rRPI-2, rRPI-3, and SPI) showed no significant difference in blood routine indexes compared with those in the CK. As compared with the CK, mice in the RPI and rRPI-1 groups produced significantly more TRH in their blood, whereas mice in other groups did not produce significantly more TRH. The relevant organ weights of the mice used for experimental testing were listed in Table 2. There was a decrease in thyroid/parathyroid index in mice fed with RPI and rRPI-1, as demonstrated by a decrease in thyroid weight and an increase in parathyroid weight. Additionally, RPI and rRPI-1 significantly reduced the weight of female mice’s ovaries, while RPI and rRPI-1 significantly increased the weight of male mice’s testes and prostate. The level of AST was significantly increased in the serum of RPI-fed mice, while the level of serum ALT was not significantly different among all experimental groups.

**Table 2:** Different groups' hematological parameters, TRH concentration and organ index values at the end of acute-toxicity testing

Parameters	CK	RPI	rRPI-1	rRPI-2	rRPI-3	rRPI-4	SPI
**Lymph (10^9^/L)**	2.01 ± 0.14^*^	1.69 ± 0.14^†^	1.80 ± 0.09*†	1.99 ± 0.21*	2.15 ± 0.17*	2.03 ± 0.16*	1.99 ± 0.11*
**Gran (10^9^/L)**	0.33 ± 0.11^*^	0.25 ± 0.09*	0.27 ± 0.10^*^	0.23 ± 0.07^*^	0.29 ± 0.06^*^	0.28 ± 0.09^*^	0.24 ± 0.07^*^
**Mon (10^9^/L)**	3.81 ± 0.82^*^	3.04 ± 0.77^*^	3.31 ± 0.95^*^	3.09 ± 0.47*	3.56 ± 0.65^*^	3.72 ± 0.49*	3.26 ± 0.63^*^
**WBC (10^9^/L)**	6.49 ± 0.27^†^	7.01 ± 0.36*	6.92 ± 0.16^*^	6.78 ± 0.19^*†^	6.56 ± 0.31†	6.51 ± 0.11^†^	6.38 ± 0.23^†^
**RBC (10^12^/L)**	6.88 ± 0.61^*^	7.36 ± 0.85^*^	6.70 ± 0.54^*^	7.13 ± 0.33^*^	7.21 ± 0.72^*^	7.47 ± 0.75^*^	7.31 ± 0.73^*^
**PLT (10^9^/L)**	556.38 ± 33.27^*^	568.92 ± 39.21*	534.96 ± 27.14*	548.89 ± 39.73^*^	562.72 ± 37.67^*^	551.56 ± 39.91^*^	560.29 ± 44.14*
**MCH (pg)**	17.17 ± 0.86^*^	16.64 ± 0.62*	16.88 ± 0.73^*^	18.02 ± 0.95*	17.66 ± 0.71^*^	17.92 ± 1.02*	18.15 ± 1.92^*^
**TRH (pg/mL)**	22.77 ± 1.96^†^	25.52 ± 2.37^*^	24.95 ± 2.44^*^	23.01 ± 2.44^*†^	22.13 ± 1.07†	22.54 ± 1.88^†^	22.08 ± 2.15^†^
**Heart (g/100 g BW)**	0.38 ± 0.01^*^	0.39 ± 0.01^*^	0.40 ± 0.02^*^	0.39 ± 0.02*	0.41 ± 0.02^*^	0.40 ± 0.01^*^	0.39 ± 0.01^*^
**Spleen (g/100 g BW)**	1.60 ± 0.22^†^	1.67 ± 0.19*	1.56 ± 0.11^*^	1.61 ± 0.14^*^	1.59 ± 0.18^*^	1.61 ± 0.18^*^	1.64 ± 0.21^*^
**Lung (g/100 g BW)**	1.68 ± 0.25^*^	1.74 ± 0.20^*^	1.71 ± 0.29^*^	1.70 ± 0.22*	1.69 ± 0.19^*^	1.79 ± 0.21^*^	1.77 ± 0.17^*^
**Liver (g/100 g BW)**	5.41 ± 0.60^*^	5.62 ± 0.59^*^	5.67 ± 0.41^*^	5.46 ± 0.72^*^	5.78 ± 0.45^*^	5.70 ± 0.55*	5.68 ± 0.62^*^
**Kidney (g/100 g BW)**	1.99 ± 0.22^*^	2.04 ± 0.29*	2.15 ± 0.18^*^	1.92 ± 0.27*	1.90 ± 0.28^*^	1.91 ± 0.24^*^	2.12 ± 0.31^*^
**Thyroid gland (g/100 g BW)**	0.10 ± 0.01^*^	0.08 ± 0.02†	0.08 ± 0.01^†^	0.11 ± 0.01^*^	0.10 ± 0.02^*^	0.09 ± 0.02*	0.10 ± 0.01^*^
**Parathyroid glands (g/100 g BW)**	0.08 ± 0.01^†^	0.10 ± 0.01^*^	0.09 ± 0.01^*†^	0.08 ± 0.01†	0.08 ± 0.01†	0.07 ± 0.01^†^	0.08 ± 0.02^†^
**Ovary (g/100 g BW)**	0.26 ± 0.06^*^	0.19 ± 0.02^‡^	0.21 ± 0.04^‡^	0.22 ± 0.03^†‡^	0.25 ± 0.03^*^	0.26 ± 0.06^*^	0.24 ± 0.04^*^
**Uterus (g/100 g BW)**	2.20 ± 0.11^*^	2.14 ± 0.09*	2.09 ± 0.15^*^	2.23 ± 0.21*	2.11 ± 0.17^*^	2.21 ± 0.12*	2.14 ± 0.19^*^
**Testis (g/100 g BW)**	0.16 ± 0.01^†^	0.21 ± 0.01^*^	0.21 ± 0.01^*^	0.19 ± 0.01^†^	0.18 ± 0.02^†^	0.17 ± 0.01^†^	0.18 ± 0.01^†^
**Prostate gland (g/100 g BW)**	0.18 ± 0.01^‡^	0.23 ± 0.02^*^	0.21 ± 0.02^*^	0.20 ± 0.01^†^	0.19 ± 0.02‡	0.19 ± 0.01^‡^	0.19 ± 0.02^‡^
**AST (U/L)**	128.83 ± 10.57†	143.75 ± 14.41*	131.19 ± 19.72^†^	127.74 ± 17.78^†^	135.54 ± 16.65^†^	130.31 ± 13.68†	131.15 ± 17.72†
**ALT (U/L)**	61.13 ± 7.72^*^	59.94 ± 6.36*	60.75 ± 7.33^*^	59.10 ± 4.34*	58.84 ± 6.02^*^	59.92 ± 5.52*	60.09 ± 4.98^*^

ALT, alanine aminotransferase; AST, aspartate aminotransferase; Gran, Number of neutrophils; Lymph, Number of lymphocytes; MCH, mean corpuscular hemoglobin; Mon, Number of monocytes; PLT, platelet count; RBC, red blood cell count; TRH, thyroid-releasing hormone; WBC, white blood cell count. The data were presented with mean ± SD, and each independent experiment was repeated three times. Different symbols of data in the same row represent significant differences at the 5% level (*P*<0.05).

## Discussion

Foods are often exposed to radioactive rays, which can affect their ingredients. In the case of cellulose, irradiation treatment will first affect the long carbon chain, and when the dose is sufficient, the carbon chain will be severed, affecting its viscosity, antibacterial activity, and mechanical strength [20]. In the present study, when the irradiation dose was increased, the RSC particles gradually became smaller and relatively uniform, which is probably caused by cellulose degradation. Further, we dissolved RSC and rRSCs in sodium hydroxide solution, and found that rRSCs and sodium hydroxide solution formed a suspension with low viscosity that was relatively clear. On the other hand, RSC formed a relatively viscous suspension (Supplementary Figure S2).

We also monitored the chemical composition of RSCs and rRSCs and found that the highest dose of irradiation (9 kGy) reduced rRSCs (rRSC-4) crude fiber content significantly. As crude fiber degradation reduces rRSC viscosity and increases solubility, more rRPI can be isolated from rRSCs as compared with RPI isolated from RSCs, thus improving the protein recovery in rRSCs [21,22]. A 32.23 ± 1.48% protein recovery yield was obtained by alkali-soluble acid precipitation method in this study, while 39.08 ± 3.01% protein recovery yield was obtained by alkali-soluble acid precipitation method after 9 kGy irradiation treatment.

There was a variation in the yield of protein recovery from RSC protein extracted by different methods in the existing research. Rommi et al. obtained a 28% protein recovery yield by alkali-dissolving and acid-precipitation, and by adding enzyme hydrolysis and other processes, the recovery yield could reach 41–53% [23,24]. While we chose the most common alkali-dissolved-acid precipitation method to obtain RPI in the present study, the recovery rate of 32.23 ± 1.48% was still higher than that in previous studies. The pH value of 10.5 was used in the present study instead of 10.0 in the previous study, and a higher pH was usually considered more conducive to protein dissolution [1]. Moreover, we chose a higher centrifugation speed (10000 ***g***) when separating the precipitate after dissolving RSC with sodium hydroxide solution. Residual solids probably contained solubilized protein, which could not be recovered from the extract. Therefore, in low-water content products, more effective centrifugation practices could facilitate protein recovery by reducing sediment entrapment of soluble components (including soluble protein) [23]. In the present study, it was confirmed that irradiation treatment of RSC can significantly improve the protein recovery yield of subsequent RPI. RSC became more soluble in sodium hydroxide solution after irradiation because part of the cellulose was degraded. As a result, the RPI’s dry matter content increased. Some of the originally bound proteins would be released after cellulose degradation, resulting in a higher protein content in the final isolation. There may be a correlation between these two changes and with the higher recovery yield of RSC proteins after irradiation.

Furthermore, fat is one of the most sensitive components to radiation. A radiation’s energy can easily change the structure of the fat’s active methylene. For example, because of irradiation, the carbon element in –CH = CH–CH or –CH = CH will undergo dehydrogenation reactions, resulting in oxidation reactions, which are also considered to be major aspects of irradiation affecting food ingredients [25]. However, it was found that irradiation treatment did not alter fat content in RSCs and rRSCs in the present study. It is noted that the irradiation dose in the present study is low, up to 9 kGy, which is generally considered to be a safe dose for food irradiation [14]. Nevertheless, food is an organic combination of multiple chemical components, and its irradiation differs from the irradiation of an individual component. A combination of different ingredients in a food will have a protective effect on one another, so its overall chemical composition will change less when it is exposed to irradiation than if it were to be exposed to irradiation alone [26]. Accordingly, we observed only a slight effect after irradiation on crude fiber, while no obvious effect was observed with respects to the content of protein, ash, or fat. It would be beneficial to pay more attention to research on low-temperature irradiation, hypoxic irradiation, or optimization of irradiation time and dose in the future to minimize the effects of irradiation on food.

In assessing a drug’s toxicity, the change in mean body weight is a key indicator. When experimental animals lose >10% of their body weight in a short period of time, a test substance is likely to have adverse effects [27]. It is possible to find a correlation between weight gain and mice’s growth status and diet. As can be seen from the body weight changes of these mice, the RPI, rRPIs and SPI groups had no statistically significant differences in body weight changes when compared with the CK. The body weight of seven groups of mice showed a normal growth trend within 14 days after administration of this study's acute toxicity testing. According to the data, these proteins have no obvious effects on mice’s growth status and food intake. Furthermore, we showed that there was no gender effect on the toxicity of RPI, rRPIs, and SPI groups. According to Finch et al. [28], the use of both genders is more ethical, as it would need less breeding to produce the numbers of mice needed for experiments.

Blood is highly sensitive to toxic compounds, and corresponding indicators can accurately reflect the physiological and pathological status of the animals [29]. The number of white blood cells in mice’s blood significantly increased after RPI was administered intragastrically, while the number of lymphocytes decreased. This change is likely due to the inflammatory response induced by intragastric administration of RPI in mice [30]. Based on the results of our mice study, along with the increased organ index of the parathyroid gland and decreased index of the thyroid gland, we concluded that RPI could lead to thyroid inflammation. Additionally, we found that the content of TRH in the RPI group was significantly increased, suggesting that the mice’s thyroid function had been impacted. Thyroid dysfunction affects the endocrine system, which affects animal reproduction [31]. There was a significant change in the weight of the ovaries, testes, and prostates of mice in the RPI group, indicating that RPI may adversely affect mice reproduction. However, all test indicators of rRPI-3 and rRPI-4 groups were normal in comparison with rRPI, indicating that irradiation treatment can indeed detoxify RSC protein in this study.

GSL found in *Brassicaceae* plants are generally considered to be the main culprits for animal feed toxicity. There has been some evidence that high level of GSL can lead to several toxic effects such as enlargement of the thyroid, reduction of plasma thyroid hormone levels, liver and kidney damage, growth retardation, decreased reproductive performance, and even death [32]. The degree of reproductive impairment is affected by animal type and glucosinolate content in existing studies. According to Martins et al. [33], GSL below 120 μmol/kg of body weight per day did not cause antinutritional or toxicity in rats, but at higher concentrations, adverse effects were observed for some GSL. Besides, pregnant female rats are sensitive to GSL, and low GSL levels have been shown to result in impaired vigor and reduced offspring survival, for example, in diets containing low-GSL rapeseed meal [34]. As compared with rats, mice were shown to have a ‘higher tolerance’ for the degradation products that may arise from glucosinolates (isothiocyanates, thiocyanate, and nitriles) [35]. Additionally, pigs are sensitive to GSL, while ruminants are less sensitive [34]. According to Lee et al. [36], the pH of the pigs' hindgut and the parent GSL composition have an impact on the composition of GSL breakdown products. Nevertheless, considering mice are the most widely used animal model in biomedical research, in this study, we assessed the impact of acute dietary exposure to RSCs in laboratory mice.

In this study, mice fed with RPI and low-dose (3 kGy)- irradiated RPI (rRPI-1) developed thyroid damage, changes in serum TRH, and reproductive system damage (ovary, testis, and prostate damage). Our hypothesis is that inflammation of the thyroid could have contributed to the negative results observed in this study. It was concluded that high-dose (7 and 9 kGy)-irradiated RPIs (rRPI-3 and rRPI-4) had a lower or no toxicity, with a median lethal dose of >20 g/kg body weight in mice. The long-term toxicity of rRPIs must be further investigated, since only a few factors were significantly different in this experiment in terms of blood system and organ indexes. While rRPI did not exhibit acute liver or kidney toxicity, prolonged toxicology experiments are still required to validate its potential as a food source for protein.

As a result of the growing concern about the detrimental effects of these antinutritional compounds on humans/animals and animal products in recent years, stricter pretreatment requirements have been introduced for meal preparation to detoxify, neutralize, or destroy undesirable compounds before feeding. ANFs come in various types, and different removal/reduction methods are often required for each type. This makes reducing/removing ANFs a relatively complex process. For example, phytic acid and polyphenol reduction can be achieved by adding alcohol to the precipitate of RSC protein during extraction [9,37]. The addition of excessive phytase to RSC can completely remove the phytic acid in the cake. In irradiation treatment, high-energy rays act on sensitive groups (e.g., –C = C–, –OH) of substances, changing their physical and chemical properties [20,38]. Phytic acid and phenolic acids contain hydroxyl groups and are sensitive to radiation. Therefore, irradiation can reduce phytic acid and phenolic acid content even at low irradiation doses.

GSL is a well-studied class of secondary metabolites that are closely related to the plant’s defense system. To provide plants with an optimal defense strategy against insects and other predatory creatures, GSL shows spatial and temporal differences in plants [32,39]. This difference requires the function of transporters and enzymes (proteins). In this study, irradiation treatment altered the properties of the proteins, directly affecting the levels of GSL in RPI. It is also possible that the irradiation energy directly interacts with the GSL molecules, and that this energy transfer can excite the electrons in the GSL molecules, resulting in chemical bond breaks, leading to degradation. However, further research is needed to understand the mechanism in greater detail.

In general, the industrial production of RSC protein products primarily rely on salt-aided ultrafiltration combined with membrane separation techniques. This process is recognized for its environmental friendliness; however, it is operationally complex and relatively expensive, which could present a disadvantage in competing with soybean isolate protein in the market [37]. On the other hand, the traditional alkaline solubilization and acid precipitation methods are simpler and more cost-effective. Nevertheless, they exhibit lower targeted extraction, resulting in protein products with comparatively inferior sensory quality and physicochemical properties [40]. Specifically, these products are characterized by a bitter taste, yellowish color, and poor solubility. Therefore, an irradiation-based optimization approach, built upon the foundation of the traditional alkaline solubilization and acid precipitation method, holds significant potential for improving the quality of RSC protein.

Overall, the strengths of our study include a significant improvement of the protein recovery yield upon irradiation treatment of RSC. The combination of radiation treatment, alkaline solubilization, and acid precipitation technique has the potential to significantly enhance the quality of RSC protein. It was discovered to be an efficient way to lower the amount of anti-nutritional elements, such as GSL, in RSC. Acute toxicity to mice, particularly to the thyroid and reproductive systems, was not demonstrated by the irradiated RSC. However, a chronic or sub-chronic study is warranted.

## Conclusion

In the present study, we treated RSC with irradiation, measured its anti-nutritional factor content, protein recovery rate, and conducted an acute toxicological analysis in mice. The aim was to find a method that can further enhance the potential of RSC as an edible protein source food. Irradiation treatment was found to effectively reduce the content of ANFs in RSC, including GSL, phytic acid, and phenolics. The irradiated RSC did not exhibit acute toxicity to mice, especially to the thyroid and reproductive systems. However, the limitations of a 14-days acute toxicity study lie in its relatively short duration, which may not capture any potential delayed or cumulative effects of the tested RSCs. Hence, a 6-month chronic animal toxicity test becomes imperative to ensure a more thorough evaluation of sustained exposure effects of the RSCs. On the basis of irradiation treatment, combined with an improved alkaline solution acid precipitation method, a higher protein recovery rate was obtained without significant changes in other nutritional components. Therefore, with our method, the utilization value of RPI can be vastly improved and a solid foundation can be laid for RPI’s final output.

## Supplementary Material

Supplementary Figures S1-S2

## Data Availability

All data can be obtained by contacting Dr Jun Song, the head of Biotechnology and Nuclear Technology Research Institute (http://www.chinawestagr.com/swjshjsyjs/).

## Abbreviations

ALT: alanine aminotransferase
ANF: antinutritional factors
AST: aspartate aminotransferase
FAO: Food and Agriculture Organization of the United Nations
FTIR: Fourier transforms infrared
GSL: glucosinolates
IAEA: International Atomic Energy Association
RPI: rapeseed protein isolate
RSC: rapeseed cake
SDS-PAGE: sodium dodecyl sulfate-polyacrylamide gel electrophoresis
SEM: scanning electron microscope
SPI: soya bean protein isolate
TRH: thyroid-releasing hormone
WHO: World Health Organization
