# Supplementary material for: Enhancing the potential of rapeseed cake as protein-source food by γ-irradiation
Source: Biosci Rep. 2024 Mar 13;44(3):BSR20231807. doi: 10.1042/BSR20231807 (PMC10938193; doi:10.1042/BSR20231807)
Supplement: Supplementary Figures S1-S2 [file BSR-2023-1807_supp.pdf]

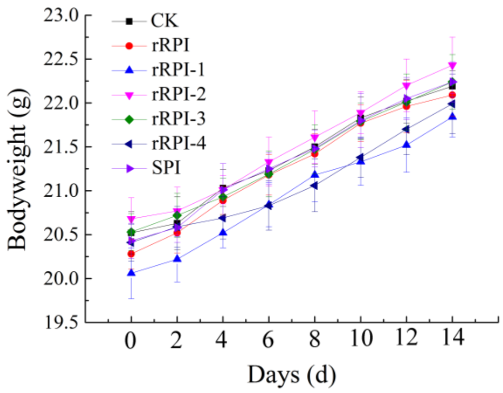

Figure S1 Bodyweight change in mice over after the acute-toxicity test in different treatment groups. Each group of mice was given different kinds of protein isolate by gavage, with a standard of 20 g/kg. RPI: Protein isolate extracted from Rapeseed cake (RSC), using alkaline solution and acid precipitation method. rRPI-1, rRPI-2, rRPI-3, and rRPI-4: Protein isolate extracted from rRSC-1 (RSC powder obtained after irradiation treatment, 3 kGy), rRSC-2 (5 kGy), rRSC-3 (7 kGy), and rRSC-4 (9 kGy), respectively. SPI, soya bean protein isolate. The data was presented with mean ± SD, and each independent experiment was repeated three times.

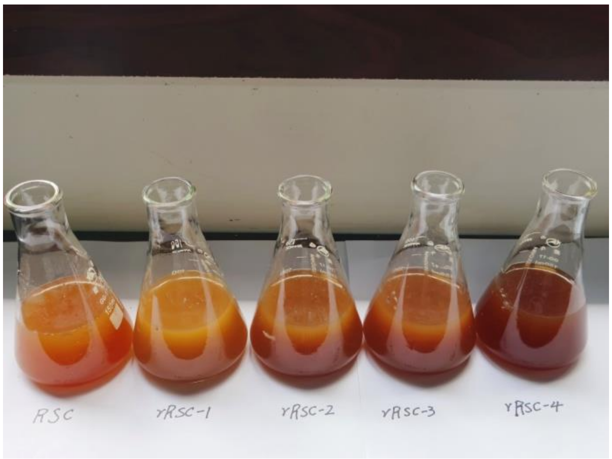

Figure S2 Color and viscosity differences between RSC and rRSCs dissolved in sodium hydroxide solution. RSC and rRSCs were dissolved in sodium hydroxide solution, rRSCs and sodium hydroxide solution formed a suspension with low viscosity that was relatively clear. On the other hand, RSC formed a relatively viscous suspension.
